# Diagnosis of Diastolic Dysfunction in Adults With Failing Fontan Circulation

**DOI:** 10.1016/j.jacadv.2025.101758

**Published:** 2025-05-14

**Authors:** Ibrahim M. Khayyat, Maria Victoria Ordonez, Ariane Marelli, Judith Therrien

**Affiliations:** a Adult Cardiology, McGill University Health Center, Montreal, Quebec, Canada; b Heart Failure and Heart Transplant Department, McGill University Health Center, Montreal, Quebec, Canada; c MAUDE Adult Congenital Cardiology Department, McGill University Health Center, Montreal, Quebec, Canada; d MAUDE Adult Congenital Cardiology Department, Department Director and Program Director, McGill University Health Center, Montreal, Quebec, Canada

**Keywords:** diastolic dysfunction, heart failure with preserved ejection fraction, failing Fontan circulation

## Abstract

Since the initial Fontan procedure introduced in 1968 for tricuspid atresia, significant advancements have expanded its application to various congenital cardiac anomalies where a biventricular circulation is unattainable. Despite improved survival rates, Fontan circulation tends to fail over time leading to late morbidity and mortality. Diastolic dysfunction is increasingly recognized as a significant contributor to circulatory insufficiency and failure in Fontan patients. This review aims to assess the current evidence for diagnosing diastolic dysfunction in adults with failing Fontan circulation, including biomarkers, echocardiography, cardiac magnetic resonance imaging, and catheterization. While advancements have been made in understanding diastolic dysfunction in single ventricles, challenges remain due to the unique anatomy and physiology of Fontan patients. Future research should focus on refining diagnostic parameters and exploring potential therapies tailored to the distinct needs of this population.

## Highlights

• Diastolic dysfunction is known as a contributor to Fontan circulatory insufficiency or failure.
• Diastolic dysfunction in Fontan patients leads to worse prognosis and worse long-term clinical outcomes during adulthood.
• Diastolic dysfunction is usually occult in the setting of a Fontan circulation, masked by the underfilled state of the single ventricle. Therefore, its prevalence is still unclear due to the challenges that carry its diagnosis.
• Diagnosing FFC due to diastolic dysfunction is feasible through a combination of noninvasive methods including biomarkers, echocardiography, and cardiac MRI, as well as invasive techniques such as cardiac catheterization.

In 1968, Fontan and Baudet introduced the initial Fontan procedure, employing total right heart bypass through atriopulmonary connection in a patient with tricuspid atresia.^1^ This surgical intervention diverts systemic venous return directly into the pulmonary circulation, allowing for passive filling without ventricular propulsion. Consequently, intracardiac mixing is eliminated, arterial saturations are increased, and volume overload on the single ventricle is reduced.^2^ The procedure results in elevated central venous pressure and diminished cardiac output (CO) due to limited preload filling of the systemic ventricle in the absence of a pulmonary pump. Over time, this procedure has been extended to a wide range of congenital cardiac anomalies where a biventricular circulation cannot be established.^2^

**Figure 1:**
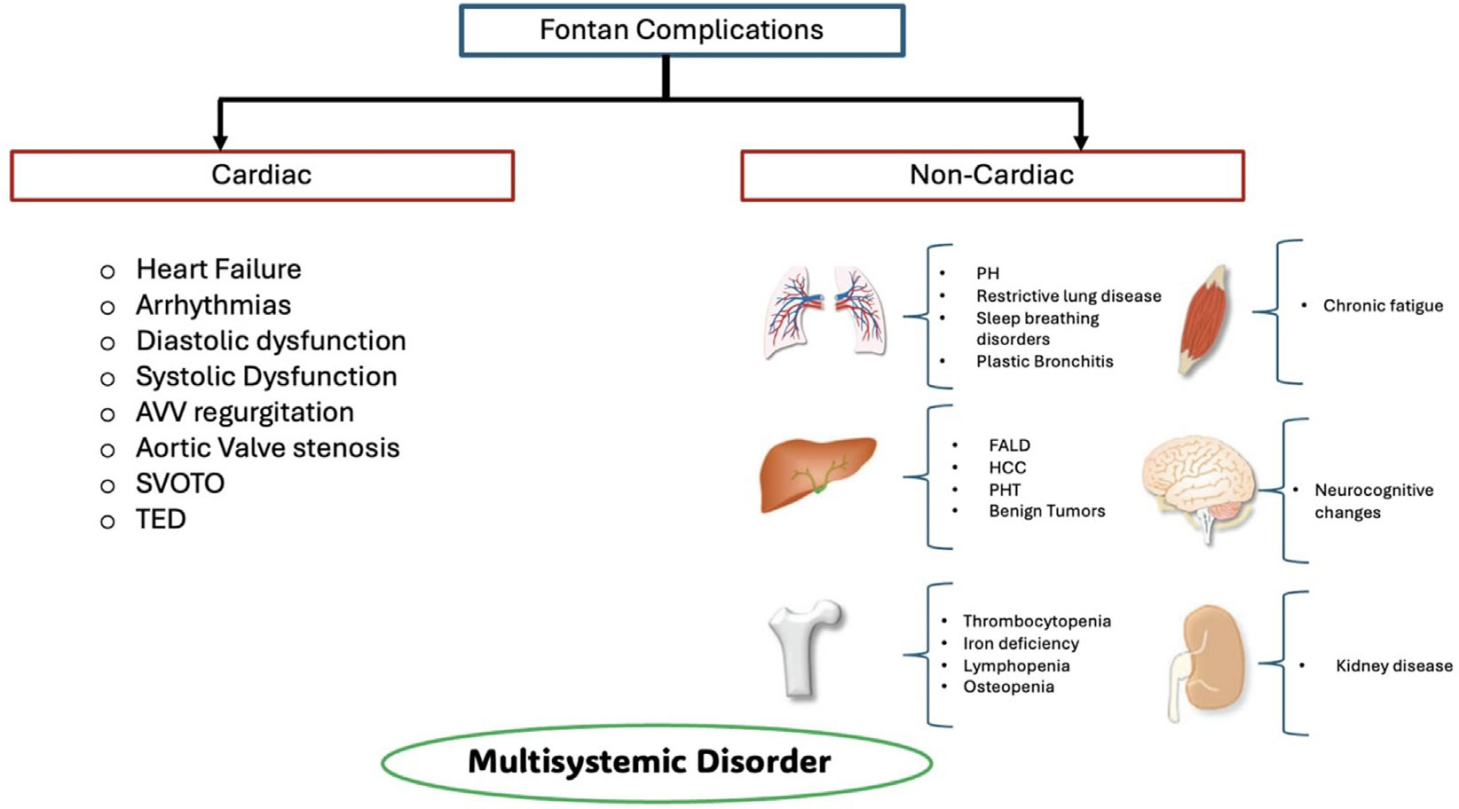
Fontan Complications AVV = atrioventricular valve; FALD = Fontan associated liver disease; HCC = hepatocellular carcinoma; PH = pulmonary hypertension; PHT = portal hypertension; SVOTO = single ventricle outflow tract obstruction; TED = thromboembolic disease.

By 2018, the global population of patients with Fontan circulation had grown to an estimated 50,000 to 70,000, with 40% being over 18 years old.^3^ The approximate 30-year survival rate after surgical completion of the Fontan procedure stands at around 85%.^4^ However, despite significant advancements since the inception of the Fontan procedure, these patients remain at heightened risk of late morbidity and mortality.^5^ Although, the Fontan palliation restores circulatory physiology, it does not correct anatomic abnormalities which leads to various cardiac or noncardiac complications (Figure 1) including ventricular dysfunction, heart failure (HF), and ultimately, the need for heart transplantation or death.^6^, ^7^, ^8^

**Figure 2:**
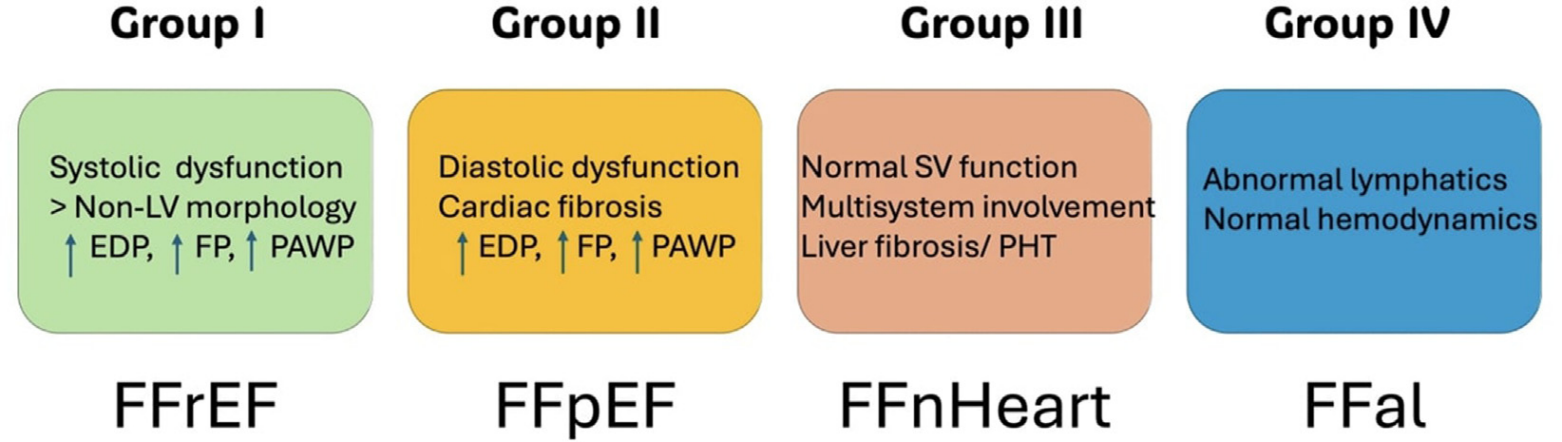
Failing Fontan Phenotypes EDP = end-diastolic pressure; FFal = failing Fontan with abnormal lymphatics; FFpEF = failing Fontan with preserved ejection fraction; FFnHeart = failing Fontan with normal heart; FFrEF = failing Fontan with reduced ejection fraction; FP = Fontan pressure; PAWP = pulmonary artery wedge pressure; SV = single ventricle.

Failing Fontan circulation (FFC) has been classified into 4 clinical phenotypes: FFC due to systolic dysfunction (group 1), FFC due to diastolic dysfunction (group 2), FFC with normal hemodynamics (group 3), and FFC with lymphatic disorders (group 4) (Figure 2).^9^

**Figure 3:**
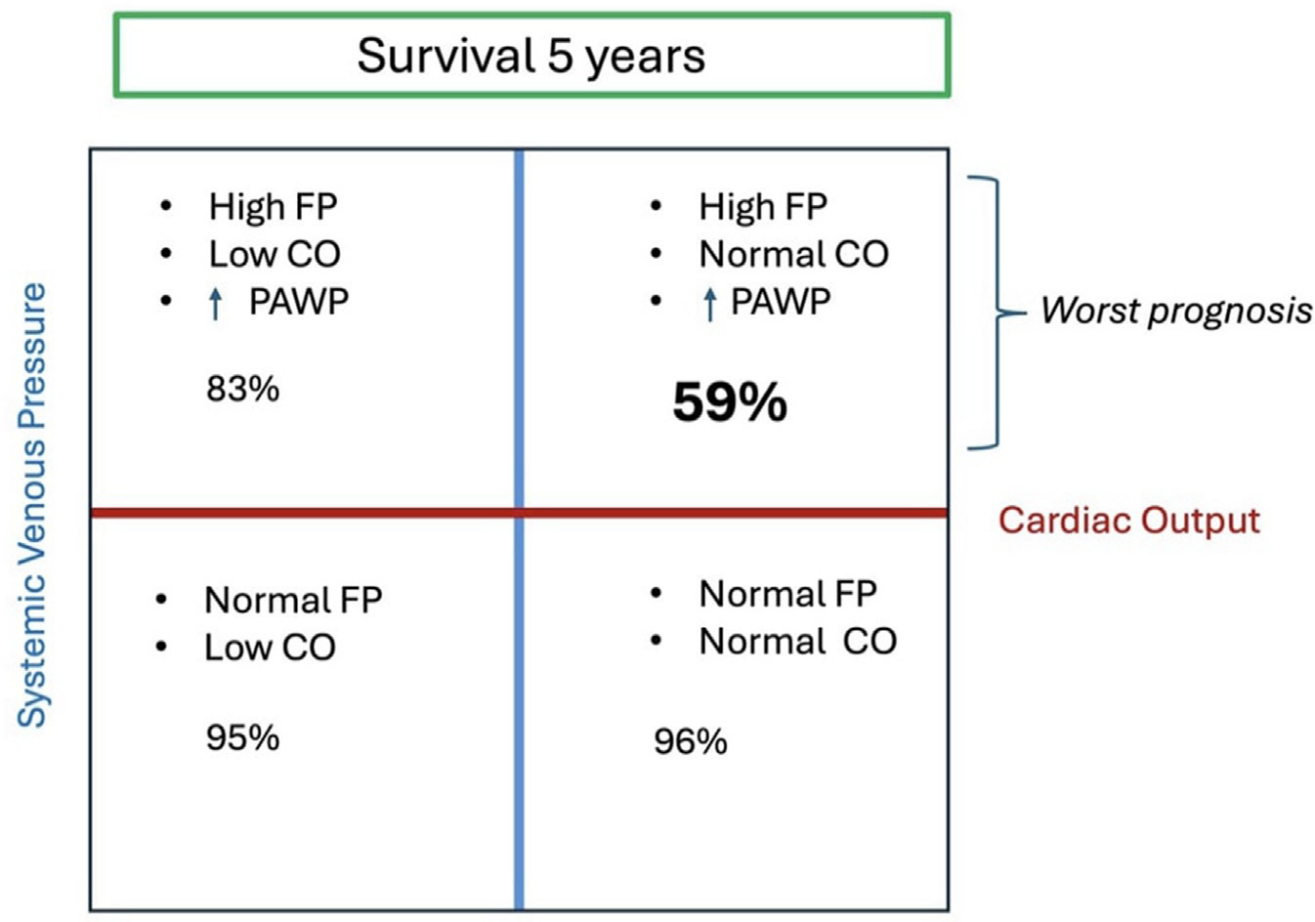
Failing Fontan Hemodynamics Scenarios and Its Correlation With Survival CO = cardiac output; other abbreviations as in Figure 2.

Miranda et al^10^ also classified adult Fontan patients with features of Fontan failure, based on their hemodynamic profile, demonstrating important prognostic implications (Figure 3).

Diastolic dysfunction is increasingly recognized as a significant contributor to Fontan circulatory insufficiency or failure.^11^ However, its physiopathology and diagnosis is still unclear. In this review, we aim to characterize and review the available evidence for the assessment and diagnosis of FFC due to diastolic dysfunction in adults with Fontan circulation.

## Pathophysiology of failing Fontan circulation due to diastolic dysfunction

Potential factors contributing to diastolic dysfunction in single ventricles include dyssynchrony, geometry, scarring or fibrosis, pre-Fontan volume overload, subsequent decreased ventricular preload, and hypoxia.^4^^,^^12^ Chronic volume deprivation without fiber stretch may decrease contractility and increase muscle stiffness, leading to elevated filling pressures.^12^ The chronically deprived single ventricle in this situation shares similarities with heart failure with preserved ejection fraction (HFpEF) pushed at its extremes.

Gewillig et al compared the Fontan circulation to a bottleneck with an upstream represented by the pulmonary impedance which hampers venous return through the pulmonary vascular bed, leading to congestion upstream and restricted flow downstream. Therefore, there is permanent reduction in CO that fluctuates based on the pulmonary vascular impedance instead of the ventricular contractility. In this situation, the ventricle is no longer controlling the CO, nor can it decrease the extent of congestion in the systemic veins; the role of the ventricle is reduced to pump the output allowed by the Fontan. However, the ventricle can (and will in time) deteriorate as a result of the limited flow through the bottleneck by increasing its end-diastolic pressure (EDP), which will worsen systemic venous congestion and further reduce output. In addition, throughout the Fontan procedure, the ventricle is exposed to extreme loading conditions, from an overloaded and overstretched ventricle to a rapidly unloaded, deprived and underfilled ventricle.^13^

These changes in size and volume during the Fontan palliation and the chronic consequences of the Fontan physiology lead, in some patients, to a stiff ventricle and an increase in the end diastolic pressure.^14^ This maladaptive univentricular remodeling can progress to ventricular failure, and ultimately death or need for transplantation.^14^

## Prevalence of failing Fontan circulation due to diastolic dysfunction

HFpEF is prevalent in the general population, accounting for a significant portion of HF cases and associated with substantial morbidity and mortality.^15^ In the failing Fontan population, HF is one of the most common cardiac complications that these patients face during adulthood.^7^ As mentioned earlier, FFC could present with different clinical phenotypes, each with its own different clinical implication. The prevalence of FFC due to diastolic dysfunction is still unclear due to the challenges that carry its diagnosis. While guidelines exist for diagnosing diastolic dysfunction in adults with HFpEF,^16^^,^^17^ diagnosing it in adult Fontan patients remains challenging.

## Clinical presentation of the failing Fontan circulation

**Figure 4:**
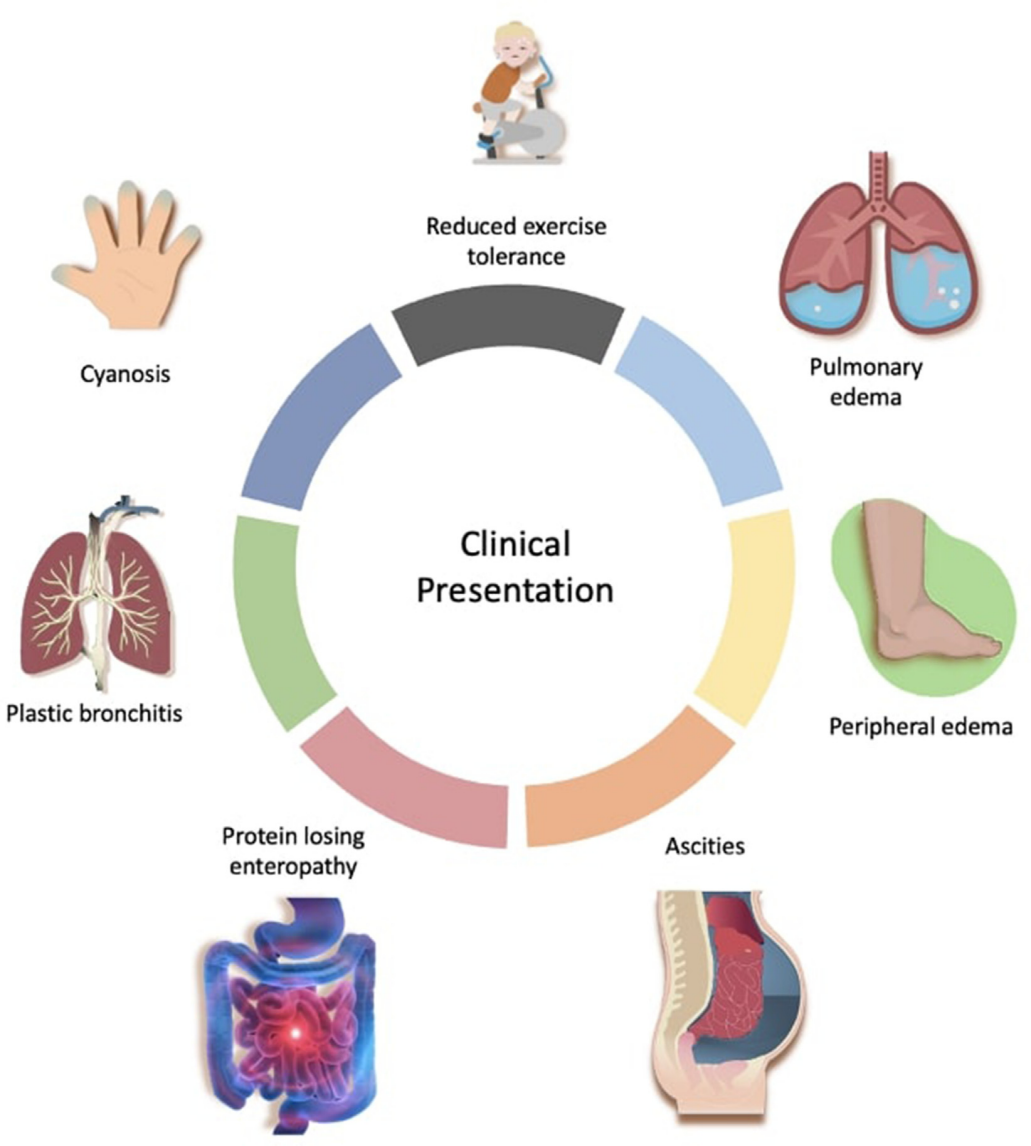
Clinical Presentation of Patients With Failing Fontan Circulation

A widely accepted definition of HF is the heart's inability to meet both resting and exercise demands at low filling pressures. Under this definition, virtually all individuals with Fontan circulation experience a physiological form of chronic HF from the outset, even though this circulation can sustain life for many years.^4^ The criteria for identifying failure in Fontan circulation differ from those used for other cardiovascular conditions. Clinical signs of Fontan circulatory failure vary widely and can include progressive jugular and hepatic venous congestion, peripheral edema, ascites, protein-losing enteropathy, plastic bronchitis, cyanosis, and exercise intolerance (Figure 4).^8^^,^^18^, ^19^, ^20^, ^21^, ^22^ However, relying solely on symptoms may not be sensitive enough for early detection of a failing Fontan circuit as many adolescents and young adults with FFC and multi-organ dysfunction do not complain of symptoms.^4^^,^^23^ Studies have shown that clinically stable patients with Fontan palliation exhibit only half the exercise capacity of controls with normal biventricular circulation.^24^ Many of these patients may not perceive such limitation and may report greater functional capacity than can be objectively demonstrated through exercise testing. Some experts suggest that a 25% decrease in maximal oxygen consumption during follow-up exercise testing is as significant an indicator of a FFC as the onset of symptoms.^25^ Notably, the underlying cause of the FFC cannot be distinguished based on clinical presentation.

## Diagnosis of the failing Fontan circulation due to diastolic dysfunction

### Role of biomarkers in diagnosis failing Fontan circulation with diastolic dysfunction

Patients with chronic Fontan circulation typically exhibit mildly elevated plasma hemoglobin levels during the stable chronic phase, alongside a relatively low platelet count likely due to liver dysfunction and hypersplenism resulting from portal hypertension.^26^ Hyponatremia and hyperuricemia are also frequently observed, reflecting the overall severity of HF.^27^ Total bilirubin and γ-glutamyltransferase levels in plasma are usually elevated in proportion to liver congestion (40%-60%), whereas liver enzyme levels (alanine aminotransferase and aspartate aminotransferase) often remain within the normal range and plasma cholesterol levels are low.^26^

In acquired heart diseases, activation of cell signaling systems, such as activation of natriuretic peptides and the renin-angiotensin-aldosterone system, occurs in response to ischemia or abnormal cardiac distension resulting from pressure or volume loading.^28^ Although specific activation pathways in congenital heart disease are less understood, elevated biomarkers indicate similar activation. Brain natriuretic peptide (BNP) has been extensively studied in congenital heart disease, with elevated serum levels observed in patients with poorer cardiovascular function or prognosis.^28^

BNP and N-terminal pro–B-type natriuretic peptide have garnered significant interest over the past 2 decades. These hormones, synthesized and released by ventricular myocytes in response to pressure overload, volume expansion, and increased myocardial wall stress, exert natriuretic, diuretic, and vasodilatory effects. Both markers demonstrate comparable clinical utility in assessing cardiac impairment and are established markers of HF in the general population.^29^

Studies have shown that BNP values in asymptomatic Fontan patients after completion of the Fontan procedure are similar to those of healthy age-matched controls. However, symptomatic patients, defined as those with NYHA functional class ≥2, exhibit significantly higher BNP levels than asymptomatic patients.^30^ A single-center study found that in acute decompensated HF, BNP levels were lower in Fontan patients compared to non-Fontan patients, 390.9 (±378.7) pg/mL vs 1,245.6 (±1,160.7) pg/mL, respectively, but higher in Fontan patients with systemic ventricular systolic or diastolic dysfunction compared to those with normal systemic ventricular function, 833.6 (±1,547.2) pg/mL vs 138.6 (±134.0) pg/mL.^26^ There is also evidence that elevated plasma BNP levels in asymptomatic Fontan patients correlate with evidence of diastolic dysfunction,^31^^,^^32^ suggesting their potential use as early markers in this population. Although there are data regarding natriuretic peptide values in patients with biventricular HF that aid in making or ruling out the diagnosis,^33^ similar cutoffs are lacking for Fontan patients. We believe the optimal approach to BNP/N-terminal pro–B-type natriuretic peptide use in this population is to establish a baseline right after the Fontan procedure and to monitor this baseline over time. It is important to note that initial elevations in asymptomatic Fontan patients with seemingly normal systolic ventricular function might indicate diastolic dysfunction.

In adults with biventricular HFpEF, serum markers of fibrosis and inflammation have been shown to be elevated.^34^ Similarly, in Fontan patients with evidence of diastolic dysfunction, elevated markers such as matrix metalloproteases and tissue inhibitor of metalloproteinase have been observed (defined as >75th percentile), suggesting their possible use as adjuncts to diagnosing diastolic dysfunction. It remains unclear if these elevated markers are the cause or consequence of myocardial fibrosis.^35^

Urinary neutrophil gelatinase-associated lipocalin, a marker of early renal dysfunction, is elevated in patients with biventricular HFpEF.^36^ Katz et al^37^ demonstrated that elevated urinary neutrophil gelatinase-associated lipocalin levels (>50 ng/mL) were associated with higher Fontan pressure, higher ventricular end-diastolic pressure (VEDP), and lower cardiac index in patients with Fontan circulation, although data regarding systolic function were lacking.

### Role of imaging in the diagnosis of failing Fontan circulation due to diastolic dysfunction

#### Echocardiography

Left ventricular diastolic dysfunction is a well-known condition in adults with biventricular HFpEF and established guidelines exist for its diagnosis using echocardiography.^16^ Parameters such as mitral valve and pulmonary vein pulsed-wave Doppler and mitral annular tissue Doppler provide valuable insights into diastolic function and have shown good accuracy in predicting increased left VEDP and pulmonary artery wedge pressure (PAWP).^16^ Furthermore, diastolic dysfunction diagnosed via echo-Doppler has a prognostic value in various cardiac disorders.^38^ However, assessing diastolic function noninvasively in patients with single ventricles can be challenging due to the lack of normal values for systemic right ventricles and univentricular hearts.

In a prospective study by Margossian et al, pediatric Fontan patients underwent transthoracic echocardiography to assess diastolic dysfunction. The diagnostic criteria were adapted from adult echocardiography guidelines with some modifications. While most patients showed evidence of diastolic dysfunction based on the proposed criteria, there was no clinically significant association found. The authors concluded that the methodology developed for assessing diastolic function in adults with biventricular hearts may not be applicable to pediatric single-ventricle patients.^39^

**Figure 5:**
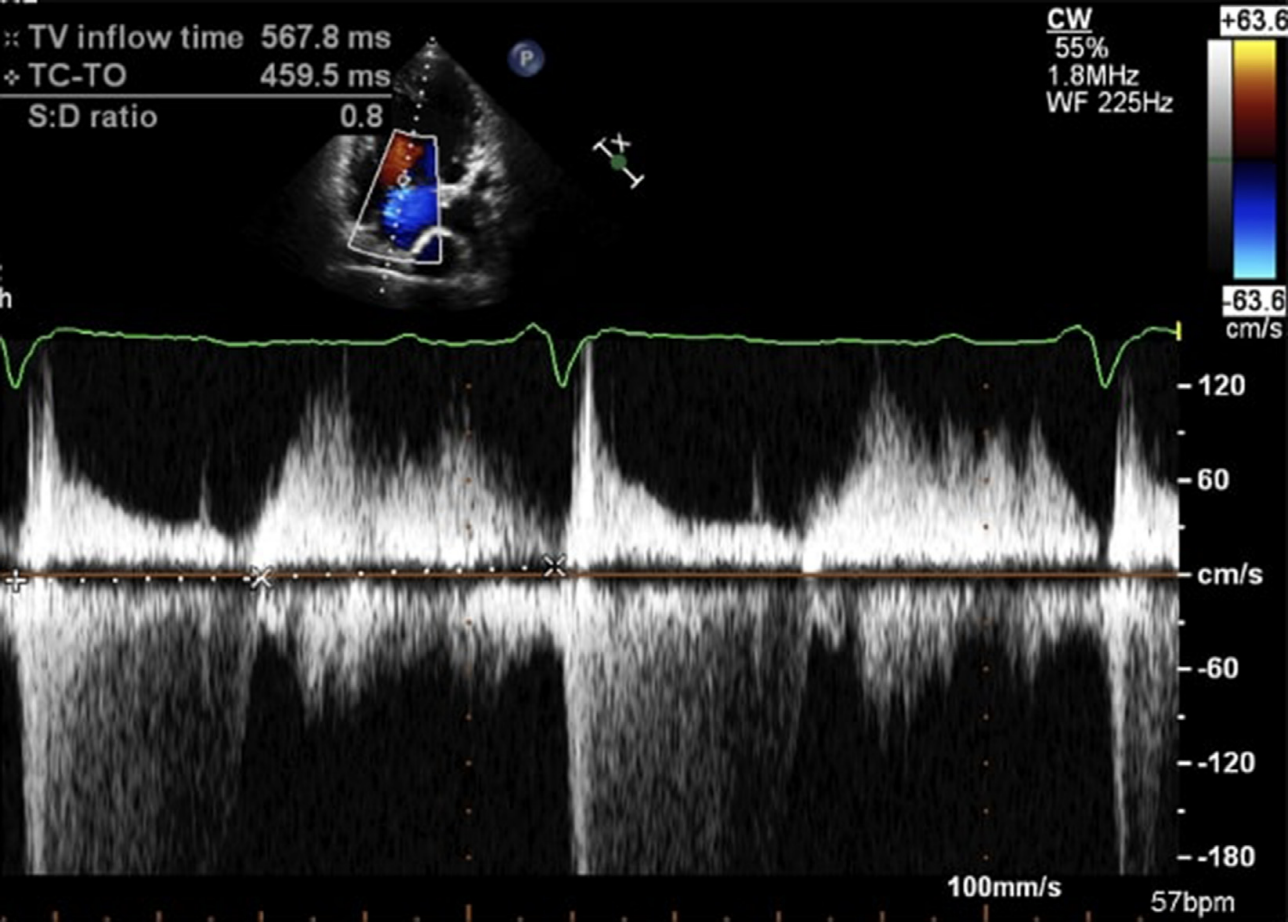
Measurement of the Atrioventricular Systolic to Diastolic Duration Ratio From the Continuous Wave Doppler Signal The atrioventricular systolic to diastolic duration ratio is calculated from the continuous wave Doppler signal of the dominant atrioventricular valve. S:D = systolic to diastolic duration; TC-TO = tricuspid valve closure to tricuspid valve opening; TV = tricuspid valve.

Reprinted from Cordina et al^40^ with permission from Elsevier. Copyright © 2024, Elsevier.

Cordina et al proposed using the atrioventricular valve systolic-to-diastolic duration ratio (AVV S/D ratio) measured with continuous-wave Doppler as a predictor of VEDP pressure in adult Fontan patients. They found that an AVV S/D ratio ≥1.1 had a 100% positive predictive value and 92% negative predictive value for detecting VEDP >10 mm Hg and was also associated with poorer peak exercise capacity (Figure 5).^40^ The same group reported in a prior study that AVV S/D > 1.1 is an especially poor prognostic marker in a relatively large group of Fontan adults.^41^

In a retrospective study of 45 adult Fontan patients with normal systolic univentricular function, Miranda et al^42^ demonstrated that atrioventricular E velocities correlated with ventricular filling pressures and that an E velocity >75 cm/s identified patients with abnormal filling pressures with good sensitivity. A short deceleration time (<135 ms) and E/A ratio >1.7 also identified patients with elevated filling pressures.^42^ This deceleration time is shorter than the deceleration time typically used to predict elevated filling pressures in patients with acquired cardiac disease (160 ms).^16^ Similarly, the optimal E/A ratio cutoff for the prediction of elevated PAWP derived from their data differs from the recommended cutoff for patients with acquired cardiac disorders.^16^ This highlights that cutoffs typically used in patients with two-ventricle circulation should not be extrapolated to Fontan patients, given their complex anatomy and physiology. Despite the good sensitivity and excellent negative predictive value of these parameters in excluding elevated filling pressures, their positive predictive values were modest.^42^ In both studies, no remarks were made regarding differences in diastolic function assessment between morphological left and right ventricles given the small number of participants.

In biventricular hearts, left atrial strain has been shown to closely correlate with left ventricular diastolic function and serves as a strong predictor for adverse cardiovascular events.^43^^,^^44^ Additionally, left atrial strain has been inversely associated with mean pulmonary artery occlusion pressure and left VEDP in such patients.^45^ However, these correlations remain unexplored in adult Fontan patients. A study by Veldtman et al investigated the relationship between atrial mechanics and invasive hemodynamic parameters in 39 pediatric Fontan patients compared to healthy controls. The study found no significant link between atrial strain and systemic ventricular filling pressures, including pulmonary artery occlusion pressure, direct left atrial pressure, or systemic VEDP. The authors noted several challenges to measuring atrial strain in Fontan patients, such as the absence of an intact interatrial septum, distortion of the right atrium due to the Fontan conduit, and the complexity of pulmonary vein connections and atrial appendages, making atrial tracing difficult.^46^

#### Cardiac magnetic resonance

In adults with biventricular HFpEF, assessing diastolic function through cardiac magnetic resonance (CMR) imaging is feasible, although it has not yet been integrated into routine clinical practice.^47^ CMR offers various indices of left ventricular diastolic function, analogous to those obtainable through echocardiography. These include parameters such as left ventricular mass and hypertrophy, left atrial size and function, profiles of mitral inflow and pulmonary venous velocity, and myocardial deformation imaging using strain.^47^ CMR also has the capability to provide tissue characterization and early detection of interstitial fibrosis, measured by T1 mapping and extracellular volume (ECV) fraction.^47^ Excessive alteration of the extracellular matrix is considered a significant contributor to the impaired cardiac relaxation and stiffness that is characteristic of HFpEF.^48^

In an observational study by Alsaied et al, 25 adult and adolescent Fontan patients with normal systolic univentricular function who underwent cardiac catheterization to assess right and left heart hemodynamics at rest and post rapid volume expansion (stress hemodynamics) were identified. Cardiac magnetic resonance imaging (MRI) with T1 mapping was used subsequently to measure ECV. Patients with ECV levels exceeding 28% (more than one SD above the mean institutional normal value) were classified as having diffuse myocardial fibrosis (DMF). Significant correlations were observed between DMF, cardiac ECV levels exceeding 28%, and elevated Fontan pressure as well as elevated EDP. A stronger correlation was noted between ECV and stress Fontan pressure and stress EDP.^35^ This study underscores the potential of CMR as a promising noninvasive method for assessing diastolic dysfunction in Fontan patients.

**Figure 6:**
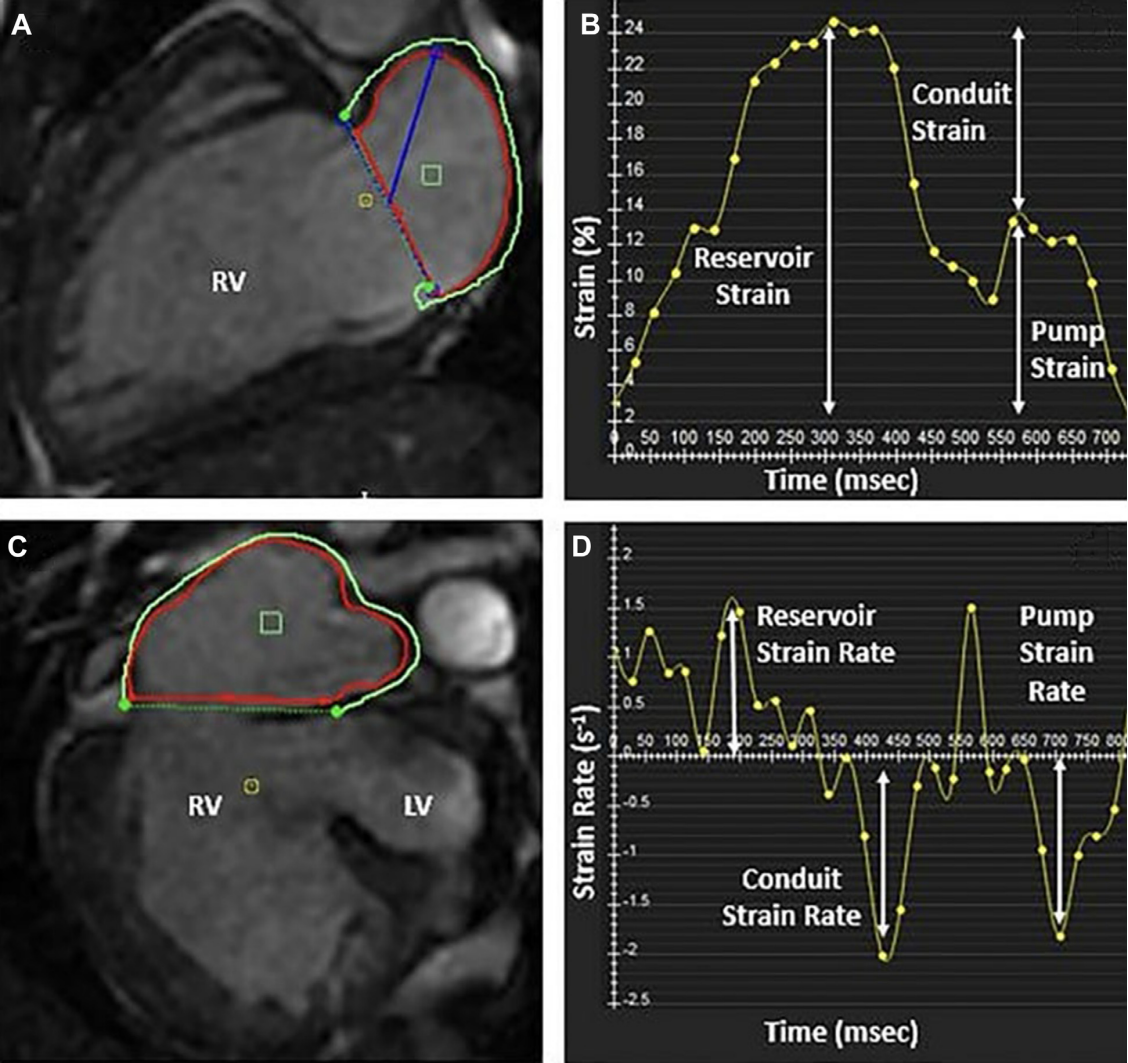
Atrial Strain Measurements in Fontan Patients Using CMR Common atrial contour measurements on (A) 2-chamber view and (C) 4-chamber view. (B) Demonstrates atrial global longitudinal strain curve. (D) Demonstrates atrial strain rate curve. Atrial conduit strain was measured by calculating the difference between reservoir and pump strain. CMR = cardiac magnetic resonance; RV = right ventricle; other abbreviation as in Figure 2.

Reprinted from Critser et al^49^ with permission from Springer Nature. Copyright © 2024, Springer Nature.

The relationship between atrial strain and diastolic function using CMR in Fontan patients was evaluated. In a study by Paul et al, 33 adult and adolescent Fontan patients underwent atrial strain measurement via CMR, and these results were compared with invasive hemodynamic data. The study found that Fontan patients exhibited reduced atrial reservoir and conduit strain but maintained atrial pump strain when compared to age-matched controls. No significant differences were observed between patients with dominant right or left ventricles or different Fontan types. More importantly, atrial conduit strain of <10% predicted an EDP ≥12 mm Hg with a sensitivity of 67% and specificity of 83% (AUC: 0.74; *P* = 0.04).^49^ Unlike echocardiography, CMR allowed accurate tracing and measurement of the common atrium and its associated structures (Figure 6).

### Role of hemodynamics in the diagnosis of failing Fontan circulation due to diastolic dysfunction

#### Catheterization

Right heart catheterization during exercise is recognized as the gold standard for diagnosing biventricular HFpEF by professional societies.^17^^,^^50^ In patients with normal ventricular systolic function, a pulmonary capillary wedge pressure (PCWP) of ≥15 mm Hg at rest or ≥25 mm Hg at any point during supine exercise or a maneuver aimed at increasing left ventricular filling pressures confirms the diagnosis of HFpEF.^51^^,^^52^ In patients undergoing upright exercise, if the increase in PCWP relative to the increase in CO (PCWP/CO) exceeds 2 mm Hg/L/min, the presence of HFpEF is confirmed.^52^

Miranda et al described the invasive exercise hemodynamics of 24 consecutive symptomatic adults who had undergone Fontan palliation and were compared with HFpEF patients and individuals with noncardiac dyspnea. Fontan patients showed lower absolute PAWP values on average than HFpEF patients, both at rest and during peak exercise, but higher values than individuals with noncardiac dyspnea. Despite similar CO at rest, CO at peak exercise was lower in younger Fontan patients compared to those with HFpEF and noncardiac dyspnea. Additionally, flow-normalized PAWP trajectories (PAWP/CO slope) were similarly steep in Fontan patients and HFpEF patients, indicating relevant diastolic dysfunction of the palliated single ventricle. The authors suggest that the lower PAWP values observed in Fontan patients may be due to the absence of a pulsatile subpulmonary ventricle and larger functional atria.^53^ In line with existing expert consensus, they propose a PAWP of ≥12 mm Hg at rest, rather than ≥15 mm Hg, as diagnostic of elevated ventricular filling pressures in Fontan patients.^54^ Another study found that a PAWP >12 mm Hg was associated with increased all-cause and cardiac mortality in adults post-Fontan.^55^ While it might seem logical to use a lower cutoff for abnormal exercise PAWP in Fontan patients compared to those with two-ventricle circulation (≥25 mm Hg), further research is necessary to establish such a threshold. Based on current available evidence, it is reasonable to accept that an end-expiratory mean PAWP at peak exercise of ≥25 mm Hg during supine exercise, roughly corresponding to a respiratory-averaged mean PAWP >20 mm Hg, may indicate relevant diastolic dysfunction with prognostic implications. A PAWP/CO slope >2 mm Hg/L/min may further support the diagnosis.^56^

**Figure 7:**
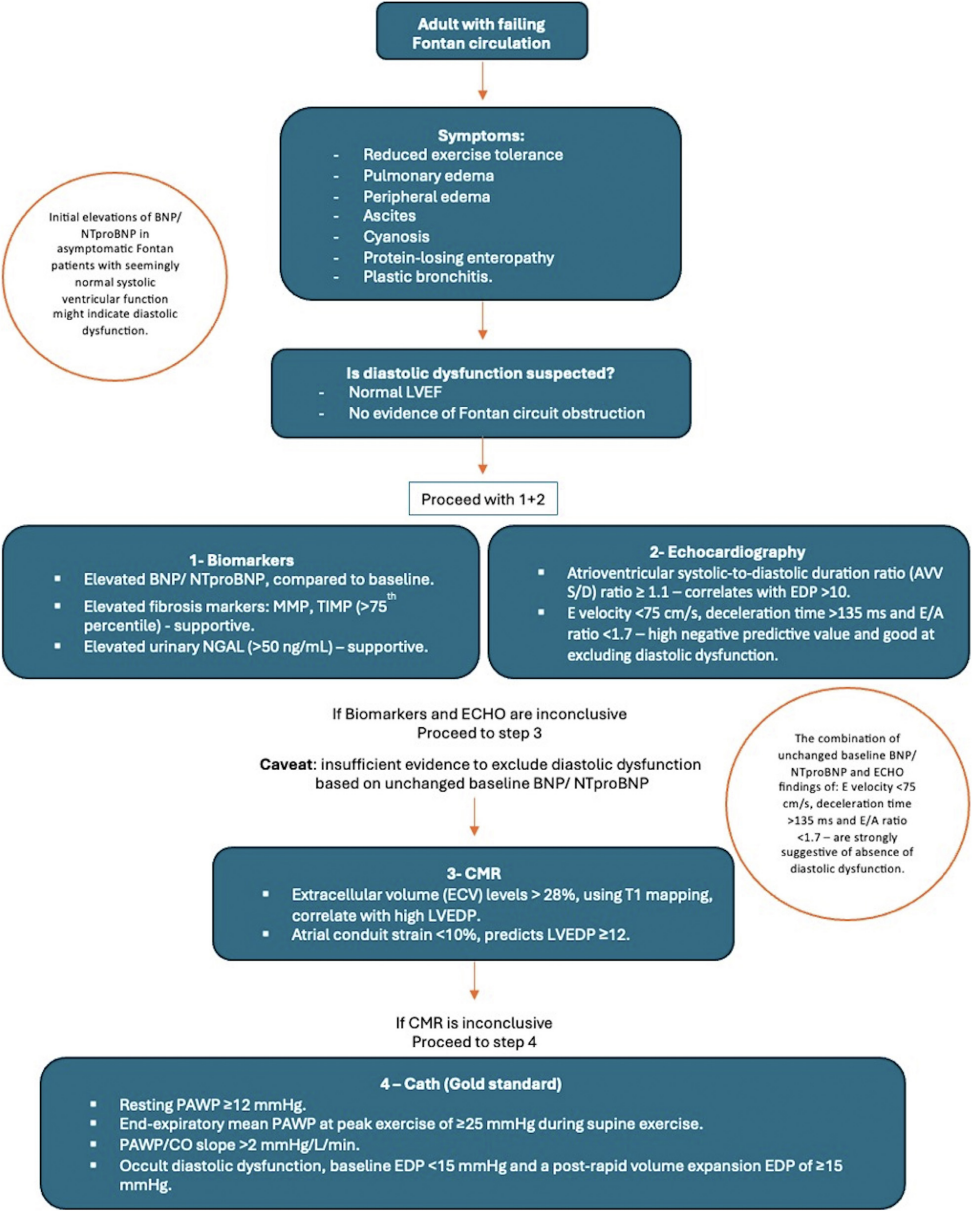
Proposed Algorithm to Diagnosing Diastolic Dysfunction in Adults With Failing Fontan Circulation BNP = brain natriuretic peptide; LVEDP = left ventricular end-diastolic pressure; LVEF = left ventricle ejection fraction; MMP = matrix metalloproteases; NTproBNP = N-terminal pro–B-type natriuretic peptide; TIMP = tissue inhibitor of metalloproteinase; urinary NAGL = urinary neutrophil gelatinase-associated lipocalin.

The use of rapid volume expansion to assess occult diastolic dysfunction in Fontan patients was evaluated in a study involving 46 Fontan patients with a baseline EDP <15 mm Hg. Rapid volume expansion involved administering a 15 mL/kg bolus of normal saline rapidly (<5 min) via central venous access, followed by a 5-minute equilibration period before repeating hemodynamic assessment. Occult diastolic dysfunction, defined as a baseline EDP <15 mm Hg and a postvolume load EDP of ≥15 mm Hg, was identified in 35% of participants. While this value was based on previous investigations in noncongenital populations, establishing a specific cutoff for diastolic dysfunction in the Fontan circulation will require further research based on the relationship between EDP and longer-term clinical outcomes.^57^ Expert consensus also supports the proposed cutoff of EDP ≥15 mm Hg after rapid volume expansion to diagnose diastolic dysfunction.^54^ New evidence form Peck et al showed that occult diastolic dysfunction is associated with an increased risk of adverse clinical outcomes, specially, in those with longer Fontan duration. The authors recommended the use of routine cardiac catheterization with rapid volume expansion to reveal the incidence of occult diastolic dysfunction^58^ (Figure 7).

### Treatment and future directions

As time progresses, cardiac complications following the Fontan procedure become more prevalent, particularly affecting adult patients. Mortality rates in Fontan circulation patients surpass those of most other congenital heart defects, except for Eisenmenger syndrome.^59^ Studies comparing mortality rates to the normal life expectancy in the United States indicate that a 40-year-old patient who underwent Fontan repair faces a mortality risk akin to that of a 75-year-old individual.^59^ Another study found high long-term mortality among adult Fontan patients with diastolic dysfunction.^55^ Failing Fontan patients with preserved ventricular function who undergo cardiac transplant have worse survival compared to a similar group with impaired ventricular function, highlighting the need to improve management and timing for transplantation in this population.^6^

Managing adult biventricular HFpEF presents challenges due to its heterogeneity, primarily focusing on managing associated comorbidities.^50^ Trials such as TOPCAT (Treatment of Preserved Cardiac Function Heart Failure with an Aldosterone Antagonist)^60^ and CHARM (Candesartan in Heart Failure: Assessment of Reduction in Mortality and Morbidity)-preserved^61^ have suggested spironolactone and angiotensin receptor blockers as potential options to reduce HF hospitalizations. Notably, EMPEROR-preserved (Empagliflozin Outcome Trial in Patients with Chronic Heart Failure with Preserved Ejection Fraction) demonstrated significant benefits in using the sodium-glucose co-transporter 2 inhibitor empagliflozin, particularly in reducing HF hospitalizations.^62^ Conversely, nitrate therapy and phosphodiesterase inhibitors have not shown significant benefits in this population.^50^

Evidence regarding medical therapy for diastolic dysfunction in the FFC is scarce and often extrapolated from HF data in adults.^11^ In a cohort study of infant patients after Fontan, 10 weeks of enalapril administration did not alter baseline hemodynamics, exercise capacity, or diastolic function and there was a suggestion of a worse, short-term, exercise capacity.^63^ Use of beta-blockers has also been limited in Fontan patients with HF. A randomized controlled trial of beta-blockade in children with single ventricles illustrated that use of beta-blockers may actually be harmful in patients with a systemic right ventricle.^64^ High-dose spironolactone has shown promise in failing Fontan patients with protein-losing enteropathy.^65^^,^^66^ Given the antifibrotic effects of spironolactone,^67^ future research into its role in diastolic dysfunction with known evidence of DMF^35^ is intriguing. In a case series of 14 pediatric Fontan patients with circulatory failure and either reduced or preserved ejection fraction, the use of sodium-glucose co-transporter 2 inhibitors was generally well tolerated with possible evidence of reverse remodeling.^68^ Given the established benefit in adults with biventricular HFpEF, it would be important to assess its therapeutic potential in the FFC patients due to diastolic dysfunction. There is currently an ongoing trial assessing the safety of dapagliflozin in adults with Fontan circulation.^69^ Additionally, specific types of exercise have been associated with improved cardiac function and quality of life in Fontan patients with no clear data regarding its effect on diastolic function.^70^ After 12 weeks, exercise cardiopulmonary rehabilitation in Fontan patients was shown to increase aerobic capacity, muscular strength, and consequently increase Vo2max and improve functional capacity.^71^

Respiratory muscle strengthening may also be of benefit. Evidence shows that 6 to 12 weeks of inspiratory training with a training device improved VO_2_max and ventilatory efficiency.^72^ Exploring the hemodynamic effects of exercise on systolic and diastolic function is of great interest. Research is also being performed to develop a cavopulmonary pump that can be placed within the Fontan conduit to increase forward pulmonary flow and preload and improve outcomes, this intervention could possibly address one of the etiologies of diastolic dysfunction in this population which is chronic volume deprivation of the systemic ventricle.^73^ Earlier detection of diastolic dysfunction in Fontan patients through noninvasive methods is crucial for identifying candidates who may benefit from these future therapies.

Fontan patients with a dominant morphological right ventricle experience more adverse cardiovascular events compared to those with a dominant morphological left ventricle, including atrial arrhythmia, cardiac transplantation, and all-cause mortality.^74^^,^^75^ Future studies should aim to identify diagnostic parameters of diastolic dysfunction based on the morphology of the ventricle. In our review, none of the trials looked at that comparison given the small number of participants and the inability to make such an assessment. Future studies assessing the efficacy of HFpEF therapies in adults with Fontan diastolic dysfunction are needed, taking into account the different ventricular morphology and its associated outcomes.

## Conclusions

Diastolic dysfunction is an underrecognized entity of the failing Fontan as it portends a challenging diagnosis. These patients experience worse prognosis during adulthood. Diagnosing FFC due to diastolic dysfunction is feasible through a combination of noninvasive methods including biomarkers, echocardiography, and cardiac MRI, as well as invasive techniques such as cardiac catheterization, which remains the gold standard (Central Illustration). Although effective medical treatments for diastolic dysfunction in this population are currently lacking, ongoing research is focused on discovering future therapies refining current diagnostic parameters.

**Central Illustration:**
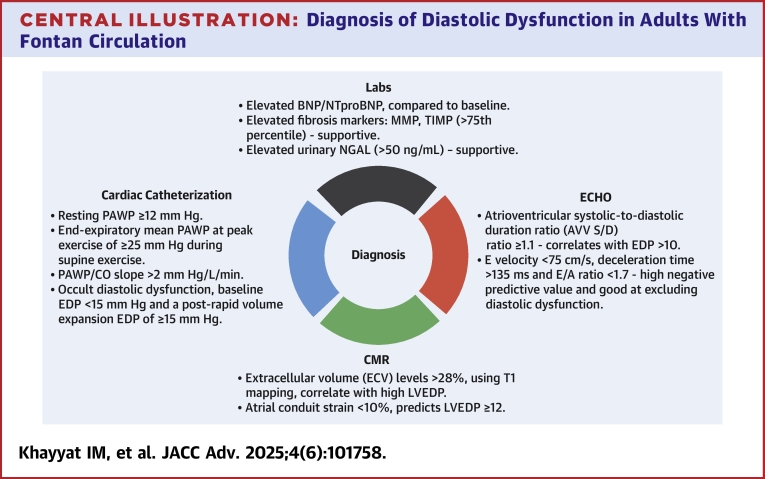
Diagnosis of Diastolic Dysfunction in Adults With Fontan Circulation Abbreviations as in Figures 2, 3, 6, and 7.

## Funding support and author disclosures

The authors have reported that they have no relationships relevant to the contents of this paper to disclose.
